# Are metformin-based combination approaches beneficial for non-small cell lung cancer: evidence from experimental and clinical studies

**DOI:** 10.1186/s40779-025-00649-5

**Published:** 2025-09-23

**Authors:** Anita Thyagarajan, Vaibhav Gajjar, Ravi P. Sahu

**Affiliations:** Department of Pharmacology and Toxicology, Boonshoft School of Medicine Wright State University, Dayton, OH 45435 USA

**Keywords:** Lung cancer, Drug repurposing, Metformin, Chemotherapy and targeted therapy, Cell signaling pathways

## Abstract

Despite having multiple treatment options, the overall outcomes, including the survival rates of non-small cell lung cancer (NSCLC) patients, remain relatively low, indicating the need to explore new approaches to achieve improved therapeutic responses. To that end, repurposed drugs such as metformin have been evaluated against many cancer types, including NSCLC. Metformin, a widely used oral hypoglycemic drug for type 2 diabetes, exhibits anticancer properties and synergy with several standards of care agents. In this review, we provide a comprehensive overview of the role and anticancer mechanisms of metformin-based combination approaches for the treatment of NSCLC. We logically discussed the experimental evidence from the in vitro and in vivo studies utilizing metformin alone, and then its combination with chemotherapeutic agents, targeted therapy, and immunotherapy. We also present clinical trials that underscore the beneficial and adverse outcomes of metformin use in combination with targeted therapy and chemotherapeutic agents, and emphasize the limitations and challenges for the treatment of diabetic and non-diabetic NSCLC patients. It appears that, regardless of the diverse anticancer mechanisms of this biguanide, the benefits may be confined to a specific patient subgroup, which opens new avenues to be explored for NSCLC treatment.

## Background

Lung cancer is one of the leading causes of death worldwide, and in the United States, it is responsible for 20 – 21% of all cancer-related deaths [1–5]. Unlike other malignancies that have static or declining rates, a persistent increase in the incidence and prevalence, as well as mortality rates, has been documented with lung cancer [5–7]. The tumor cells begin to grow within the lining of the bronchi, bronchioles, or alveoli. Pathologically, lung cancer is categorized into two types: small cell lung cancer (SCLC) and non-small cell lung cancer (NSCLC). Of all lung cancer cases, 10 – 15% represent SCLC, which has a rapid growth rate and is divided into two categories: combined small cell carcinoma and small cell carcinoma, which is also known as oat cell cancer, and most of the SCLC cases are of oat cell cancer [8–10]. The most common type of lung cancer is NSCLC, which accounts for 80 – 85% of cases and is the most prevalent subtype with a poor prognosis [11–13].

NSCLC is classified into 5 types: adenocarcinoma, squamous cell carcinoma, large cell carcinoma, adenosquamous carcinoma, and well-differentiated neuroendocrine tumor [9]. The most common form of NSCLC is adenocarcinoma, accounting for 40% of the cases, and is usually found in the outer regions of the lungs [9]. Squamous cell carcinoma is the second most common form, accounting for 30% of the cases, and originates from the bronchial tubes [13]. The third type is a large cell carcinoma (15%), which generally grows faster and begins to develop in the outside edges of the lungs [9, 14]. About 0.4 – 4.0% of diagnosed NSCLCs are adenosquamous carcinoma, which is comprised of glandular and squamous cells [9, 14]. Neuroendocrine tumors have two subtypes, typical and atypical, of which typical neuroendocrine tumors have a better prognosis and are occasionally associated with neuroendocrine syndrome [9, 14, 15]. In NSCLC, survival rates depend on the tumor site. For example, the rate of survival for localized NSCLC (which does not spread outside of the lung) is 63% and 35% for regional NSCLC (which spreads outside of the lung) [16]. Of several risk factors involved in the development of lung cancer, globally, air pollution is the second leading cause of lung cancer [17]. The other common risk factors include exposure to: 1) cigarette smoking; 2) radon gas, arsenic, chromium, beryllium, and asbestos; 3) alcohol consumption; and 4) family history [18–21].

Given the types of lung cancer and various risk factors involved, to make a treatment decision, factors such as size and location of the tumor, histologic type, margins of surgery, grade of tumor, and role of pleura have been suggested to be considered before. The most common treatment approaches are: 1) surgery with anatomic resection like sublobular resection, segmentectomy, and lobectomy [22, 23]; 2) chemotherapy using platinum and taxol-based drugs such as cisplatin and paclitaxel [24, 25]; 3) radiation therapy, e.g., stereotactic body radiotherapy (SBRT) [26, 27]; 4) immunotherapy, where immune checkpoint inhibitors like programmed cell death protein-1 (PD-1) and cytotoxic T-lymphocyte associated protein 4 are mostly used and have been associated with promising outcomes in a subset of patients [28, 29]; and 5) targeted therapy, a drug treatment focusing on specific tumor cells having aberrated signaling pathway(s). For example, there are FDA-approved treatments (e.g., erlotinib, gefitinib, and Osimertinib) for the receptor tyrosine kinases (RTKs) family members, such as epidermal growth factor receptor (EGFR), which is hyperactivated or mutated in NSCLC patients [30, 31]. In addition, other treatment settings involve both neoadjuvant and adjuvant therapy, where the neoadjuvant phase consists of preoperative chemotherapy or radiotherapy, and the adjuvant phase involves postoperative therapy, during which drugs, including investigational agents such as metformin, may be administered [32, 33].

## Drug repurposing

Drug repurposing or drug repositioning is defined as the identification of a new indication of pre-existing drugs with known mechanisms of action for a defined pathology or disease condition, including cancer [34]. The major advantage of using the repurposed drug is to reduce the timeline of investment and potential risks. For instance, with the drug reuse concept, a new drug can be developed within 3 – 12 years, whereas rudimentary procedures require up to 15 years or more to be fully developed. However, drug repositioning avoids the slow progression and side effects [35, 36]. These drugs are already approved by the regulatory bodies; thus, they have been through clinical trials and approved for human use directly with the portfolio, including dosing regimens, adverse events, drug-drug interactions, known pathways through which drugs are acting, and pharmacokinetics and pharmacodynamics parameters [37].

While several pharmacological agents have been explored for drug repurposing, we review metformin effects, as this is one of the widely used drugs that has been tested against various cancer models, including lung cancer [38]. The first evidence of metformin effects against cancer was reported by Libby et al. [39] in Tayside, Scotland (UK). In the large cohort study, a total of 13,334 diabetes patients with NSCLC were included, and the data demonstrated that metformin treatment resulted in greater cancer-free survival in the patients compared to the other group of patients treated with other drugs [39].

## Metformin

It has been more than 60 years since metformin (1, 1-dimethyl biguanide hydrochloride) was first introduced for diabetic treatment, and since then it has become one of the most prescribed hypoglycemic (i.e., glucose-lowering) drugs in the world with its potential role for therapeutic applications in the future [40]. Metformin is a synthetic derivative of guanidine extracted from Galega officinalis, also known as French lilac, goat’s rue, Italian Fitch, Professor Wood, or Spanish sainfoin [41]. Earlier, from 1920 to 1930, guanidine derivatives, including metformin, were used to treat diabetes mellitus, which was later discontinued due to the reported toxicity and availability of insulin injections. In 1940, the drug was rediscovered in search of antimalarial agents, and finally, in 1957, the proper usage of metformin in the treatment of diabetes was first reported by a French physician, Dr. Jean Sterne [42].

**Fig. 1:**
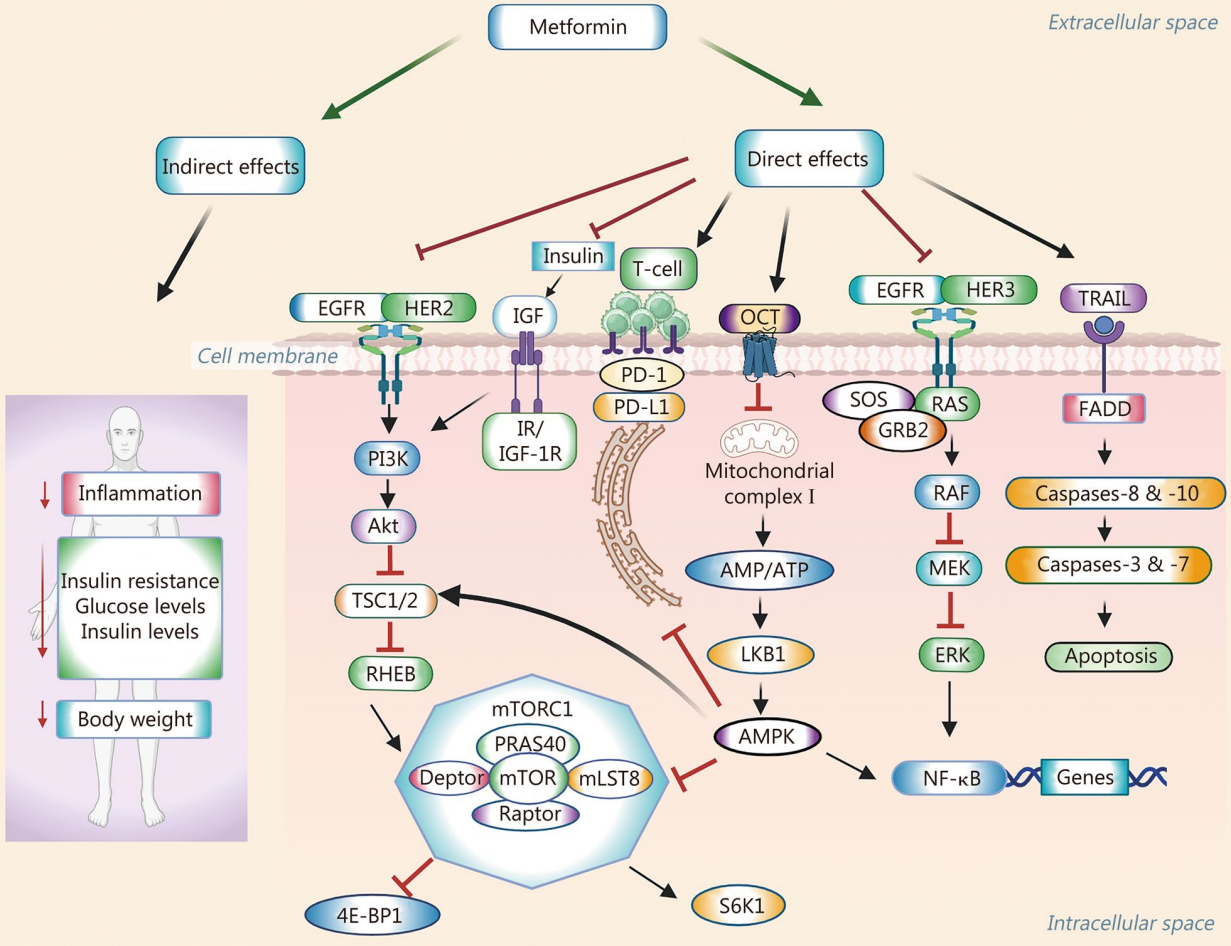
Antitumor mechanisms of metformin. Schematic representation of the indirect and direct mechanisms of metformin is presented. Akt protein kinase B, AMP adenosine monophosphate, ATP adenosine triphosphate, AMPK AMP-activated protein kinase, Deptor DEP domain-containing mTOR-interacting protein, 4E-BP1 eukaryotic translation initiation factor 4E binding protein 1, EGFR epidermal growth factor receptor, ERK extracellular signal-regulated kinase, FADD Fas-associated death domain, HER2 human epidermal growth factor receptor 2, IGF insulin growth factor, IGF-1R IGF-1 receptor, LKB1 liver kinase B1, MEK mitogen-activated protein kinase kinase, mTORC1 mammalian target of rapamycin complex 1, NF-κB nuclear factor-kappa B, OCT1 organic cation transporter 1, PD-1 programmed cell death protein-1, PD-L1 programmed cell death-ligand 1, PI3K phosphatidylinositol 3-kinase, PRAS40 proline-rich protein kinase B substrate of 40 kD, RAS rat sarcoma viral oncogene homolog, RHEB Ras homolog enriched in brain, S6K1 ribosomal protein S6 kinase B1, SOS son of sevenless, TRAIL tumor necrosis factor-related apoptosis-inducing ligand, TSC1/2 tuberous sclerosis proteins 1 and 2

Among the common mechanisms, metformin exerts its anti-cancer activity via two mechanisms: indirect and direct **(**Fig. 1**)**. The indirect mechanisms of metformin involve: 1) reduction of inflammation Due to its ability to decrease the levels of inflammatory markers such as Toll-like receptors 2 and 4, tumor necrosis factor-α, and C-reactive protein [43–45]; 2) improvement in insulin resistance by decreasing the levels of circulating insulin, and glucose levels via inhibiting gluconeogenesis in the liver and stimulating glucose uptake in the muscle [45–47]. In addition, metformin increases the levels of glucagon-like peptide-1 (GLP-1) by enhancing the expression of GLP-1 receptors in the pancreas as well as reducing the breakdown of GLP-1 by inhibiting dipeptidyl peptidase-4 activity [48, 49]; and 3) causing body weight loss via decreasing appetite [50].

Among the direct mechanisms in tumor cells, metformin binds with the organic cation transporter 1 (OCT1), which is required for its uptake in tissue. Metformin inhibits the mitochondrial complex I via increasing the ratio of adenosine monophosphate (AMP) to adenosine triphosphate (ATP), and activates liver kinase B1 (LKB1) and the AMP-activated protein kinase (AMPK) pathway, resulting in the inhibition of downstream protein complex, mammalian target of rapamycin complex 1 (mTORC1) [51, 52]. This mTORC1 constitutes the key regulators, such as proline-rich protein kinase B substrate of 40 kD, mammalian target of rapamycin (mTOR), Dishevelled, Egl-10, and Pleckstrin (DEP) domain-containing mTOR-interacting protein (Deptor), Raptor, and mLST8. The inhibition of mTORC1 induces ribosomal protein S6 kinase B1 (S6K1, involved in cell growth, survival) and inhibits eukaryotic translation initiation factor 4E binding protein 1 (4E-BP1, which regulates protein synthesis) [51, 52]. AMPK phosphorylates tuberous sclerosis proteins 1 and 2 (TSC1/2), which act as tumor suppressors and regulate cell growth and division [51, 52]. TSC1/2 inhibits a downstream Ras homolog enriched in brain (RHEB) protein that regulates cell growth, proliferation, and regeneration via the mTOR pathway [51, 52]. Nevertheless, inhibition of the mTOR pathway has been observed with metformin without its effects on LKB1, AMPK, and TSC1/2 signaling, indicating its off-target mechanisms regulating the activation of the phosphatidylinositol 3-kinase (PI3K), protein kinase B (Akt), and mTOR signaling pathways in tumor cells [53, 54].

APMK activation decreases the expression level of programmed cell death-ligand 1 (PD-L1, which acts as a brake to prevent T cells via binding to an immune checkpoint protein, PD-1, expressed on T cells), which induces cytotoxic T-cell-mediated tumor cell death [51, 55]. Moreover, other signaling cascades such as EGFR/human epidermal growth factor receptor 2 (HER2), PI3K/Akt, insulin growth factor (IGF)/IGF-1 receptor (IGF-1R, which also activates PI3K/Akt), nuclear factor-kappa B (NF-κB), EGFR/HER3, rat sarcoma viral oncogene homolog (RAS)/rapidly accelerated fibrosarcoma (RAF)/mitogen-activated protein kinase (MEK)/extracellular signal-regulated kinase (ERK), and tumor necrosis factor-related apoptosis-inducing ligand (TRAIL), Fas-associated death domain (FADD) pathways, which play critical roles in regulating cell proliferation, cell cycle, and caspase-mediated apoptosis, are identified as the common targets of metformin in cancer chemoprevention, including in NSCLC models [56–59].

Importantly, metformin’s mechanisms of action are multifaceted and differ across different histologic subtypes of lung cancer with distinct pathological and genomic contexts. For example, in EGFR-mutant lung adenocarcinoma cells, metformin inhibits mitochondrial electron transport complex 1 to induce AMPK activation, leading to the inhibition of the mTOR pathway, which causes G0/G1 cell cycle arrest, mitochondrial apoptosis, and reduction of tumor growth [60, 61]. However, in LKB1-deficient NSCLC cells, metformin restores the inactive AMPK signaling despite the absence of the canonical upstream kinase, likely through engaging an alternative pathway [53]. In tumor protein p53 gene (TP53) mutant squamous cell carcinomas, metformin exploits metabolic dependencies created by higher glycolytic activity due to increased expression of glucose transporter 1 and hexokinase 2, for its anti-cancer mechanism [62]. Unlike NSCLC, in SCLC, metformin inhibits the PI3K/Akt pathway, but paradoxically activates the MEK/ERK pathway [63]. This indicates that the limited efficacy of metformin in SCLC could be enhanced by the combination of MEK/ERK pathway inhibitors.

## Experimental evidence from the in vitro and in vivo studies with metformin or its combination with chemotherapy, targeted therapy, and immunotherapy

### Metformin

MicroRNAs (miRNAs) are single-stranded small (21 – 22 nucleotides) non-coding RNA molecules, which regulate gene expression by recruiting messenger RNAs to the miRNA-induced silencing complex, resulting in diminishing mRNA 3’ translation region through base pairing [64, 65]. Among various miRNAs, miR-7 is identified as a potential tumor suppressor via its ability to target the EGFR pathway in glioblastoma and plays a critical role in regulating such disease conditions. Considering the anti-cancer properties of metformin and its ability to target miRNAs [66, 67], Dong et. al. [68] determined the effect of metformin on miR-7 regulation using the NSCLC model. The authors demonstrated that treatment with metformin or miR-7 mimic resulted in the inhibition of cell viability, cell migration, and cell invasion in a dose and/or time-dependent manner in the A549 cell line. In addition, it was found that miR-7 expression was upregulated by metformin through the AMPK pathway, as an AMPK inhibitor significantly reduced metformin and miR-7 mimic induced increased miR-7 expression [68]. These findings suggest that a decreased level of AMPK can downregulate the level of miR-7. Interestingly, metformin and miR-7 mimic reduced protein expression of p-Akt, p-mTOR, p-ERK1/2, and p-p65, which suggests that the combination treatment would exert an enhanced effect in downregulating NF-κB, mitogen-activated protein kinase (MAPK)/ERK, and Akt/mTOR pathways. These findings suggest that metformin upregulates miR-7 through AMPK and regulates different signaling pathways to exert its anti-cancer effects [68]. The summary of in vitro and in vivo studies with metformin or its combination with other drugs is shown in Table 1 [59, 68–78].

**Table 1:** Summary of in vitro and in vivo studies with metformin, or its combination with anti-cancer agents

Study type	Mouse model	Cell line(s)	Therapeutic agent(s)	Targets/ Pathways	Findings	References
In vitro	–	A549	Metformin	miR-7, AMPK, NF-κB, Akt/mTOR, MAPK/ERK	Decreased cell viability, migration, and invasion	[68]
In vitro, in vivo	Athymic nude mice	H460, A549, H1650	Metformin	TRAIL	Increased apoptosis and decreased cell proliferation, and tumor growth	[59]
In vitro	–	A549	Metformin + 2-dDG	Glycolytic pathway, p38, and AMPK	Increased cell cytotoxicity, ROS generation, mitochondrial membrane potential, and apoptosis	[69]
In vitro, in vivo	PDX73 and PDX111 models in SCID mice	A549, H1299	Metformin + Cisplatin	KRAS/LKB1	Decreased tumor volume, increased apoptosis, and inhibition of drug resistance	[70]
In vitro, in vivo	BALB/c male mice	Cisplatin resistant A459, H838 cell lines	Metformin + Cisplatin	Nrf2 and HO-1	Overcomes chemoresistance, induces apoptosis, and decreases cell proliferation	[71]
In vitro, in vivo	Athymic nude mice	A549, H1299	Metformin + Celecoxib	p53, p21, RAF/MEK/ERK and PI3K/Akt	Decreased cell proliferation, cell migration, and increased cell apoptosis and cell cycle arrest	[72]
In vitro	–	Ncl-H2087	Metformin + Trametinib	ERK, AMPK, PI3K/Akt/ mTOR	Biphasic anti-tumor effect	[73]
In vitro	–	PC9R, PC9R/OS	Metformin + Gefitinib/Osimertinib	EGFR-TKIs resistance	Increased cell apoptosis and sensitized EGFR-TKIs-resistant NSCLC cells	[74]
In vitro, in vivo	Female BALB/c	A549, H460	Metformin + Anlotinib	AMPK/ mTOR/ACC/ HIF-1α and MAPK	Decreased cell proliferation and intracellular ATP levels, and increased cell apoptosis	[75]
In vitro, in vivo, ex vivo	Male BALB/cAnN, Cg-Foxnlnu/crlNARC	A549, H1975, HCC827	Metformin + Pemetrexed	AMPK, CDKN1B, Akt and cyclin D1	Decreased cell proliferation and angiogenesis, induced cell cycle arrest, increased apoptosis, and decreased tumor growth	[76]
In vitro, in vivo	PBMCs-CDx mouse	A549, H460	Metformin + Pembrolizumab	STING, TBK1 and TRF3 pathways	Overcome primary resistance to PD-1 inhibitors and reduce tumor growth	[77]
In vitro, in vivo	SPF nude C57BL/6 J mice	A549, H1299	Metformin + Atezolizumab	AMPK-CEBPB-PD-L1	Inhibited cell proliferation and tumor growth	[78]

*2-dDG* 2-deoxy D-glucose, *ATP* adenosine triphosphate, *miR-7* microRNA 7, *AMPK* AMP-activated protein kinase*, NF-κB* nuclear factor kappa-B, *Akt* protein kinase B*, mTOR* mammalian target of rapamycin, *MAPK* mitogen-activated protein kinase, *ERK* extracellular signal-regulated kinase, *TRAIL* tumor necrosis factor-related apoptosis-inducing ligand, *LKB1* liver kinase B1, *KRAS* Kirsten rat sarcoma viral oncogene homolog, *Nrf2* nuclear factor erythroid-2 related factor 2, *HO-1* heme oxygenase-1, *RAF* rapidly accelerated fibrosarcoma, *MEK* mitogen-activated protein kinase kinase, *EGFR-TKIs* epidermal growth factor receptor-tyrosine kinase inhibitors, *HIF-1α* hypoxia-inducible factor-1α, *CDKN1B* cyclin-dependent kinase inhibitor 1B, *STING* stimulator of interferon genes, *TBK1* TANK-binding kinase 1, *CEBPB* CCAAT-enhancer-binding protein beta, *PBMCs* peripheral blood mononuclear cells, *PD-L1* programmed cell death-ligand 1, *PDX* patient-derived xenograft, *PI3K* phosphatidylinositol 3-kinase, *TRF3* TATA-box-binding protein-related factor 3

As TRAIL exhibits an anti-cancer activity [79, 80], a study by Liu et al. [59] determined the effects of metformin on TRAIL-mediated effects using in vitro and in vivo models of NSCLC. The authors demonstrated that metformin inhibits the proliferation and survival of H460, A549, and H1650 NSCLC cell lines in a dose-dependent manner via inducing apoptosis as measured by increased cleavage of poly (ADP-ribose) polymerase (PARP), a nuclear enzyme involved in multiple cellular processes, including apoptosis, caspase-8, and caspase-3. To investigate the involvement of the TRAIL-death receptor pathway, metformin was found to induce the protein levels of TRAIL without altering the expression of TRAIL receptors, DR4 and DR5. This contradicts the hypothesis regarding the role of DR4 and DR5 in metformin-mediated apoptosis. As metformin induces TRAIL secretion, it was found that TRAIL-R2 Fc chimera protein, which is a human recombinant protein containing an extracellular domain and shortened intracellular domain of DR5 and Fc fragment of immunoglobulin G, neutralizes TRAIL’s ability to induce cell death. These findings indicate that metformin increases endogenous TRAIL expression in lung cancer, which is secreted into a conditioned medium to trigger autocrine stimulation and apoptosis via a death receptor [59]. Similar results were obtained in animal studies where reductions in tumor size and tumor growth were documented. Overall, these findings indicated that the activation of the TRAIL death receptor pathway is essential to promote the anti-cancer activity of metformin.

### Metformin and chemotherapy

**Fig. 2:**
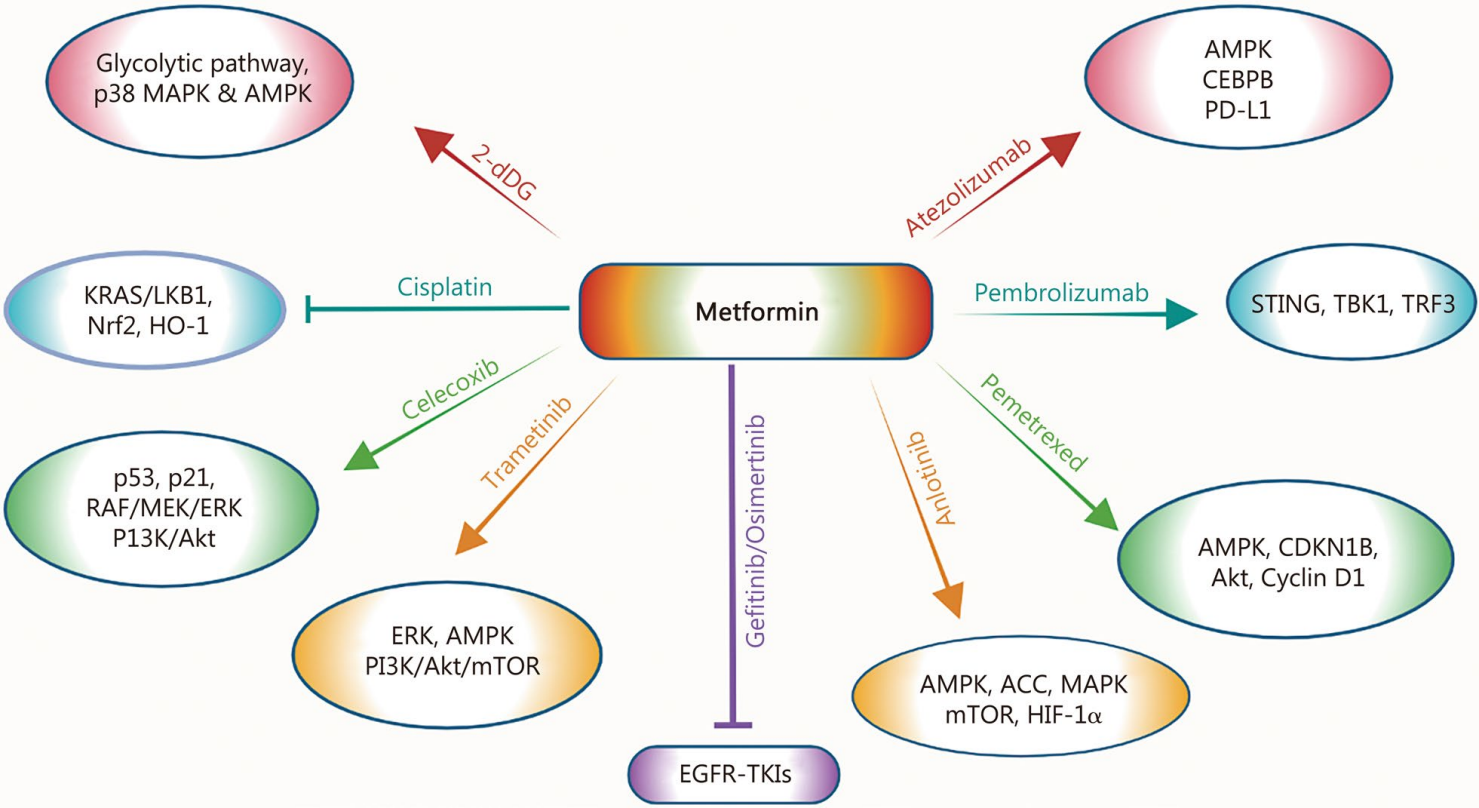
The cellular mechanisms of metformin and its combination with chemotherapy, targeted therapy, and immunotherapy for NSCLC treatment are presented. Agents with anticancer properties, 2-dDG, cisplatin, celecoxib, gefitinib/osimertinib, anlotinib, pemetrexed, pembrolizumab and atezolizumab target diverse signaling cascades (shown above) to inhibit multiple cellular properties and/or overcome resistance mechanisms (as mentioned in Table 1). 2-dDG 2-deoxy D-glucose, Akt protein kinase B, AMP adenosine monophosphate, AMPK AMP-activated protein kinase, ASS acetyl-coA carboxylase, CDKN1B, cyclin-dependent kinase inhibitor 1B, CEBPB CCAAT/enhancer-binding protein beta, EGFR epidermal growth factor receptor, ERK extracellular signal-regulated kinase, HIF-1α hypoxia-inducible factor-1α, HO-1 heme oxygenase-1, KRAS Kirsten rat sarcoma viral oncogene homolog, LKB1 liver kinase B1, MAPK mitogen-activated protein kinase, MEK mitogen-activated protein kinase, mTORC1 mammalian target of rapamycin complex 1, Nrf2 nuclear factor erythroid-2 related factor 2, PD-L1 programmed cell death-ligand 1, PI3K phosphatidylinositol 3-kinase, RAF rapidly accelerated fibrosarcoma, STING stimulator of interferon genes, TBK1 TANK-binding kinase 1, TRF3 TATA-box-binding protein-related factor 3, TKIs tyrosine kinase inhibitors

In addition to its use as a single agent, metformin has also been explored in combination with different chemotherapeutic agents [69–76]. The schematic representation of metformin mechanisms in combination with therapeutic agents is shown in Fig. 2.

#### Metformin and 2-deoxy D-glucose (2-dDG)

A study conducted by Hou et al. [69] investigated the relevance of targeting intracellular metabolism as an alternative approach to NSCLC treatment. To that end, considering the antiproliferative properties of 2-dDG that are mediated through the glycolytic pathway, the authors demonstrated that metformin and 2-dDG combination reduces the viability of A549 cells, which resulted in the induction of lactose dehydrogenase (LDH) release [69]. LDH is a cytoplasmic enzyme that is released upon damage to the plasma membrane. Besides, this combination resulted in dramatically increased levels of thiobarbituric acid, a marker of lipid peroxidation, indicating the degeneration of cell membrane lipids and induction of cell damage [69]. Additionally, compared to individual treatments, metformin and 2-dDG combination induced an increased cell death via inducing reactive oxygen species (ROS) generation and alterations in the mitochondrial membrane potential. Importantly, metformin and the 2-dDG combination resulted in the suppression of antioxidant enzymes such as superoxide dismutase, glutathione, and catalase, which process blocked by the pretreatment of antioxidant, N-acetyl cysteine [69]. While evaluating the effects of both drugs on DNA adducts and DNA damage, it was found that combination treatment induced DNA damage by increasing tail length and DNA adduct formation, as confirmed by immunohistochemical studies demonstrating the nuclear localization of 8-hydroxy-2’-deoxyguanosine (8-OHdg) [69]. Moreover, it was observed that combination therapy caused increased induction of early/late apoptosis as confirmed by caspase-3 activity, as well as increased activation of p38-MAPK and AMPK signaling pathways [69]. Overall, these findings provided the rationale for further exploration of metformin and 2-dDG combination for the treatment of NSCLC (Fig. 2).

#### Metformin and cisplatin

Notably, Kirsten rat sarcoma viral oncogene homolog (*KRAS*) is one of the frequently altered genes in NSCLC, and KRAS-activating mutations exhibit other concurrent mutations in genes, including *LKB1* [81, 82]. While the exact roles of various *KRAS* mutations remained unclear, LKB1 functions to suppress tumor cell survival, tumor growth, and metabolic activity by targeting the AMPKα1 pathway, as AMPKα1 gets activated under metabolic stress to spare energy units and nutrients [83]. As metformin induces metabolic stress, Moro et al. [70] determined the effects of metformin to selectively target *KRAS/LKB1* co-mutated NSCLC cells. The data demonstrated that metformin treatment resulted in significantly greater inhibition of cell growth and induction of apoptosis in A549 cells harboring KRAS/LKB1 commutation as compared to H1299 cells having wildtype KRAS^WT^/LKB1^WT^, in a concentration-dependent manner [70]. While metformin caused higher apoptosis in A549 cells than in H1299 cells, G1 cell cycle arrest was specifically seen in H1299. To determine the differential sensitivity of metformin-induced apoptosis in A549 vs. H1299 cells and its dependence on the *KRAS* and *LKB1* gene mutation status, as well as to evaluate metformin’s effect with cisplatin combination, the authors generated A549 *LKB1*^WT^ and H1299 cells with different *KRAS* and *LKB1* status, including *KRAS*^WT^/*LKB1*^WT^, *KRAS*^WT^/*LKB1*^del^, *KRAS*^G12C^/*LKB1*^WT^, and *KRAS*^G12C^/*LKB1*^del^. The data demonstrated that metformin and cisplatin caused increased apoptosis in A549 *LKB1*^WT^ and H1299^WT/WT^ cells with enhanced effects in cells having double mutation [70].

The in vivo studies utilized 2 patient-derived xenograft (PDX) models, PDX111 harboring KRAS^G12V^/LKB1^WT^ and PDX73 harboring KRAS^G12V^/LKB1^K287X^ mutations [70]. The data demonstrated that metformin inhibited the growth of PDX111 tumors at a lower dose, but had no effect on the growth of PDX73 tumors. However, at higher metformin dosage, tumor growth suppression was observed in both the PDXs, and that synergistic response was observed with cisplatin [70]. Importantly, the metformin and cisplatin combination targets CD133^+^ cancer stem cells in PDXs with *KRAS*/*LKB1* co-mutation to prevent resistance to cisplatin. Taken together, metformin enhanced the apoptosis and anti-cancer activity of cisplatin in NSCLC with *KRAS*/*LKB1* mutations and prevented resistance to cisplatin.

The efficacy of chemotherapeutic agents is limited due to drug resistance, mediated in part via ROS-induced oxidative stress [84–86]. Notably, hyperactivation of nuclear factor erythroid-2 related factor 2 (Nrf2), a master regulator of antioxidant defense response, has been shown to promote NSCLC development and enhance chemoresistance [87, 88]. To that end, Huang et al. [71] determined the mechanism of metformin and Nrf2 in cisplatin-induced ROS generation and chemoresistance in NSCLC cells. The data demonstrated that metformin and cisplatin combination synergistically reduced cell proliferation, enhanced apoptosis, and decreased the expression of Nrf2 and heme oxygenase-1 (HO-1), one of the antioxidant phase II detoxifying enzymes, in normal NSCLC H838 cell line, and cisplatin-resistant A549 cell line A549/DDP [71]. Further studies demonstrated that shRNA-mediated *Nrf2* knockdown increased mitochondrial ROS generation and cisplatin sensitivity and cytotoxicity of the NSCLC cells; whereas overexpression of Nrf2 and HO-1 decreased the level of intracellular ROS by metformin and cisplatin [71]. These studies suggested that the detoxification of Nrf2 and HO-1 is essential for tumor cells to acquire cisplatin drug resistance [71].

Mechanistically, cisplatin-mediated increased expression of Nrf2 and HO-1 proteins, as well as phosphorylation of Raf and ERK1/2, were reduced by metformin treatment in both H838 and A549/DDP cell lines [71]. These findings indicated that the inactivation of the RAF-ERK pathway plays a critical role in the metformin-induced decreased expression of the Nrf2/HO-1 axis via ubiquitin proteasome-mediated degradation of the Nrf2 protein. Importantly, decreased tumor growth, reduced protein expression of the Nrf2/HO-1 axis, and increased apoptosis were observed in A549/DDP tumor xenografts by cisplatin and metformin treatment compared to monotherapy [71]. Moreover, Nrf2 expression on patients’ survival after neo-adjuvant chemotherapy was evaluated using tumor samples from the NSCLC patients. Patients were classified into 2 groups: one with decreased Nrf2 expression, including patients who exhibited low Nrf2 score after neo-adjuvant chemotherapy, and the second group with non-decreased Nrf2 expression, including patients exhibiting unchanged or higher Nrf2 score. In the non-decreased Nrf2 group, the majority of NSCLC patients were associated with poor survival and chemoresistance as compared to the patients in the Nrf2 decreased group, indicating that Nrf2 expression could be used as an important clinical marker to predict the prognosis of patients undergoing cisplatin-based neoadjuvant chemotherapy [71]. Taken together, cisplatin and metformin combination reduced Nrf2 protein expression by suppressing the RAS/RAF and ERK pathways to eliminate detoxification and induce ROS-mediated apoptosis (Fig. 2).

#### Metformin and celecoxib

Along similar lines, Cao et al. [72] determined the effects of celecoxib, a nonsteroidal anti-inflammatory drug (NSAID) with anti-proliferative properties with metformin to overcome drug resistance and side effects induced by NSAIDs. The data demonstrated that the metformin and celecoxib combination resulted in decreased cell viability and lower DNA proliferation rates in A549 and H1299 cell lines, indicating that the suppression of cellular DNA synthesis inhibits cell proliferation and induces cytotoxicity, as confirmed by low LDH secretion as compared with metformin and celecoxib alone [72]. Furthermore, the combination treatment induced cell apoptosis as measured by increased cleavage of caspase-3, -7, -8, -9, and PARP as well as upregulation of pro-apoptotic proteins, Bad and Bax, and reduced expression of anti-apoptotic proteins, B-cell lymphoma-extra large (Bcl-xl) and B-cell lymphoma 2 protein (Bcl-2), indicating the activation of both endogenous and exogenous pathways in eliciting apoptotic activity [72].

To validate intracellular metabolism changes, combined treatment was found to increase the level of intracellular ROS [72]. As excessive ROS can induce DNA damage, metformin and celecoxib treatments were found to increase the levels of gamma histone H2AX (γ-H2AX), a marker of DNA damage. These Changes promoted DNA injury resulting in increased activation of ataxia-telangiectasia mutated and checkpoint kinase 2, as well as G1 phase cell cycle arrest via induced expression of p53 and p21 signaling cascades [72]. Mechanistically, inhibition of the RAF/MEK/ERK and PI3K/Akt pathways by metformin and celecoxib was found to be involved in the induction of apoptosis [72]. Moreover, the metformin and celecoxib combination inhibited cell migration and invasion by reducing the expression of N-cadherin, p-FAK, and matrix metalloproteinase 9. Furthermore, the combination treatment caused decreased growth of A549 tumor xenografts without exerting a deleterious effect on the mice’s bodyweight, as well as induced tumor necrosis and tumor apoptosis [72]. Overall, these findings indicated that the metformin and celecoxib combination exerts promising antitumor effects via their ability to target multiple cell signaling pathways (Fig. 2).

### Metformin and targeted therapy

#### Metformin and trametinib

The RAS/RAF/MEK/ERK pathway plays a central role in tumor cell proliferation and signals through receptor tyrosine kinases. A study by Ko et al. [73] determined the combined effect of metformin and trametinib, a MEK inhibitor, using NCI-H2087 NSCLC cells harboring neuroblastoma RAS viral oncogene homolog (NRAS) and v-RAF murine sarcoma viral oncogene homolog B (BRAF) mutations. These studies demonstrated that metformin treatment resulted in a dose-dependent increase in activation of ERK1/2 and AMPK and suppression of the PI3K/Akt/mTOR pathway. However, trametinib treatment exerted an opposing effect on ERK1/2 and AMPK activations, but did not substantially affect the PI3K/Akt/mTOR pathway. Interestingly, the combination of metformin and trametinib resulted in synergistic attenuation of metformin-induced increased ERK1/2 and AMPK activation as well as inhibition of cell viability and colony formation at lower doses compared to individual treatments [73]. However, at high doses, this combination caused an antagonistic effect and induced cell survival and proliferation, indicating that such a discrepancy could be attributed to the differences in the mutational status of NSCLC cells [73]. Overall, these findings indicated that a combination of metformin and trametinib results in a biphasic antitumor effect, which could be dependent on the alterations of signaling cascades, including ERK activity and mutational status of lung cancer cells.

#### Metformin and osimertinib

RTKs belong to the growth receptor family members and are responsible for tumor cell growth and angiogenesis. Thus, various inhibitors targeting RTKs [i.e., tyrosine kinase inhibitors (TKIs)] have been approved for the treatment of lung cancer [89, 90]. As drug resistance remained one of the major hurdles in limiting the efficacy of EGFR-TKIs for NSCLC treatment, a study by Han et al. [74] determined the effect of metformin in overcoming EGFR-TKI resistance using in vitro models and retrospective clinical data. For in vitro studies, the osimertinib-resistant PC9R/OR cell line was generated from gefitinib-resistant PC9R cells. Metformin treatment exerted synergistic effects and re-sensitized PC9R cells to gefitinib, and PC9R/OS cells to osimertinib. These findings indicate that metformin can overcome EGFR-TKIs resistance by sensitizing EGFR-TKI-resistant cells to EGFR-TKIs via lowering their median inhibition concentration (IC_50_) values. Besides, metformin treatment caused synergistic apoptosis induction when combined with both EGFR-TKIs [74] (Fig. 2).

#### Metformin and anlotinib

Similarly, Zhu et al. [75] determined the anti-tumor efficacy of metformin with anlotinib, an oral RTK inhibitor that targets vascular endothelial growth factor receptor (VEGFR), fibroblast growth factor receptor, platelet-derived growth factor receptor, and C-Kit [89, 91]. Metformin combination with anlotinib was found to potentiate the inhibition of cell viability and colony formation ability of A549 and H460 NSCLC cell lines compared to individual drug treatment [75]. In a xenograft model, the combined treatment also decreased tumor growth without eliciting any change in the body weight of mice. Furthermore, the combination treatment caused increased cell apoptosis via inducing BCL2-associated X protein (Bax) and decreasing Bcl-2 expression, as well as increasing caspase-3 and PARP cleavage as compared to monotherapy [75]. Moreover, metformin also induced AMPK activation and suppressed mTOR phosphorylation.

As acetyl-coA carboxylase (ACC) plays an important role in the biosynthesis and oxidation of fatty acids, the combination treatment was found to induce ACC activation, downregulate hypoxia-inducible factor-1α (HIF-1α) expression, and decrease intracellular ATP levels, resulting in the inhibition of ATP production [75]. Since HIF-1α promotes glycolysis to induce ATP production, these studies indicate that metformin and anlotinib combination targets the AMPK/mTOR/ACC/HIF-1α axis. Given that ROS plays an important role in cell apoptosis, and the oxidized nicotinamide adenine dinucleotide phosphate (NADP^+^)/reduced nicotinamide adenine dinucleotide phosphate (NADPH) ratio is considered a redox couple of oxidative stress, this combination treatment increased the intracellular NADP^+^/NADPH ratio to regulate homeostasis and promote oxidative stress [75]. This ROS-mediated cell death was found to be regulated by the MAPK pathway as metformin and anlotinib combination increased the expressions of MAPK family members, including p38, c-jun N-terminal kinase, and ERK1/2 [75]. Taken together, metformin and anlotinib combination exerts anti-cancer effects by activating AMPK/mTOR and MAPK/ERK pathways, and metformin sensitizes NSCLC to anlotinib; thus, this combination has the potential to be explored in clinical settings for malignancies, including NSCLC (Fig. 2).

#### Metformin and pemetrexed

Given that chemotherapy is the first choice of treatment when none of the driver mutations is found to be activated in NSCLC [92, 93], studies by Wang et al. [76] determined the anti-cancer effects of metformin and pemetrexed combination in NSCLC models. The data demonstrated that the metformin and pemetrexed combination significantly decreased the proliferation and colony formation ability of A549, H1975, and HCC827 cell lines. Besides, the combination treatment resulted in cell cycle arrest at the S phase and increased apoptosis, whereas a low cytotoxic effect was documented on normal Lung cells. Mechanistically, increased activation and expression of AMPK and cyclin-dependent kinase inhibitor 1B (CDKMB), as well as decreased activation and expression of Akt and cyclin D1, were involved in the regulation of cell cycle arrest [76]. In a xenograft model of BALB/cAnN mice, combination therapy reduced blood vessel density along with the length of the blood vessel network and blood vessel segments, suggesting the suppression of vessel growth and inhibition of angiogenesis [76]. Importantly, combination therapy resulted in additive inhibition of A549 orthotopic tumor growth as well as the percentage of endothelial cell markers, such as VEGF-positive area and platelet endothelial cell adhesion molecule-1 (PECAM-1)-positive area, compared to monotherapy. Moreover, reduced expression of tumor angiogenic markers such as endoglin, VEGFR, and carcinoembryonic antigen was documented by metformin and pemetrexed treatment [76]. Overall, these studies indicated that the metformin & pemetrexed combination exerts promising antitumor and antiangiogenic effects against NSCLC (Fig. 2).

### Metformin and immunotherapy

#### Metformin and pembrolizumab

Most of the NSCLC harbor mutations in a tumor suppressor serine/threonine kinase 11 (STK11), also known as the *LKB1* gene, one of the potential factors responsible for resistance to immune checkpoint inhibitors, such as PD-1 therapy [94, 95]. To investigate whether metformin could enhance the efficacy of immune checkpoint inhibitors in NSCLC harboring STK11 mutation, Wang et al. [77] conducted studies with metformin and pembrolizumab, a PD-1 inhibitor. Metformin treatment resulted in reduced cell viability and cell proliferation via increasing IFN-γ secretion by activated T cells in STK11 mutant A549 and H460 NSCLC cells. In a mouse model, a combination of metformin and pembrolizumab treatment significantly reduced the growth of H460 tumor xenografts and enhanced CD8^+^ T-cell infiltration [77]. Besides, T-cell-mediated tumor cell death by metformin was found to be mediated via increased activation of stimulator of interferon genes (STING) and downstream TANK-binding kinase 1 (TBK1) and TRF3 pathways.

Notably, AXIN-1, a protein encoded by the *AXIN-1* gene, was found to be necessary for STING activation and served as a platform for several protein functions required for binding with STING [77]. Besides, metformin combination with pembrolizumab decreased cell viability and cell proliferation in AXIN-1^+/+^ H460 cells, indicating the specific role of axis inhibition protein 1 (AZIN-1) in the stabilization of STING to induce cell death. The key amino acid of AXIN-1 is Lys 107 (K107), which binds with another residue of STING at the Lys 150 (K150) site. K107 is a composition of multiple amino acid residues, including THR-192, GLU-195, GLN-159, GLU-188, and ASN-195 [77]. Metformin binds with the GLU-195 in AXIN-1 via hydrogen bonds to induce the binding of AXIN-1 and STING at the Lys 150 site [77]. STING was found to be the target of E3 ubiquitin ligases TRIM32 and TRIM56, which are important for recruiting TBK1 for STING activation. Overall, the findings demonstrated that metformin decreases K48-linked STING ubiquitination by reducing its binding with E3 ligand RNF5 to overcome the resistance of PD-1 inhibitors in NSCLC harboring STK11 mutations (Fig. 2).

#### Metformin and atezolizumab

Lu et al. [78] demonstrated that the antitumor effect of metformin is mediated through the regulation of AMPK and CCAAT-enhancer-binding protein beta (CEBPB)- PD-L1 expressions. PD-L1 is a ligand of PD-1, and CEBPB is a transcription factor having three isoforms: liver-enriched transcriptional activator protein (LAP)*, LAP, and liver-enriched inhibitory protein (LIP), where LAP* plays an important role in the inhibition of CEBPB. Besides, the activation or inhibition of CEBPB transcription depends on the ratio of LIP/LAP. The data demonstrated that metformin treatment inhibits cell proliferation and PD-L1 expression in a dose-dependent manner in A549 and H1299 NSCLC cell lines [96]. Next studies showed that PD-L1 has a binding site for CEBPB and that metformin upregulates CEBPB expression, suggesting that CEBPB could regulate PD-L1 transcription directly by binding to its specific site. Importantly, the overexpression of LAP in A549 cells resulted in increased cell proliferation and migration as well as PD-L1 upregulation, whereas overexpression of LIP in A549 cells had opposite effects on cell proliferation and PD-L1 expression [78]. Importantly, metformin upregulates AMPKα1 activation, LAP* and LIP expression, and downregulates PD-L1 expression to exert anti-tumor effects. In addition, AMPKα1 activation significantly reduced the expression of three isoforms of CEBPB, confirming that AMPKα1 can also regulate the expression of PD-L1 and CEBPB [78]. In a mouse model, metformin treatment decreased the growth of Lewis lung carcinoma tumor growth, and an enhanced antitumor effect was observed with anti-PD-L1 (atezolizumab) immunotherapy. Overall, these results confirmed that the metformin-induced anti-tumor effect is mediated via its ability to regulate the AMPKα1/CEBPB/PD-L1 signaling pathway (Fig. 2).

## Evidence from the clinical studies with metformin or its combination with EGFR-TKIs, chemotherapy, and chemoradiotherapy

### Metformin

In addition to in vitro and in vivo studies, clinical studies have determined the efficacy of metformin with or without other therapeutic agents in NSCLC patients. To that end, Xiao et al. [97] presented a meta-analysis on the association between metformin treatment and the risk of survival in lung cancer patients with type 2 diabetes. Overall, the data demonstrated that metformin treatment was associated with significantly decreased lung cancer incidence/risk and increased survival in lung cancer (both NSCLC and SCLC) patients. Interestingly, the subgroup analysis by ethnicity indicated that metformin’s protective effect on lung cancer risk was noticed in Asian patients but not in European patients. However, the beneficial effect of metformin on lung cancer survival was documented in both Asian and non-Asian lung cancer patients.

Notably, a study by Lu et al. [78] performed a retrospective analysis on lung adenocarcinoma patients who underwent surgery, and their tumor tissues were screened for PD-L1 gene expression. Tumor cells or tumor stromal cells with a score of ≥ 1% were defined as PD-L1 positive. Similar results were also observed in clinical specimen analysis of adenocarcinoma patients, demonstrating increased expression of CEBPB and decreased PD-L1 expression in the metformin-treated group compared to the control group. The survival probability analysis demonstrated that patients with low expression of CEBPB-LAP and PD-L1 had significantly better prognoses compared to those patients with high expression of CEBPB-LAP and PD-L1 [78]. Overall, these studies indicated that CEBPB regulates PD-L1 gene expression that could be modulated by metformin to achieve a better prognosis. The summary of clinical studies is given in Table 2 [74, 78, 96, 98–101].

**Table 2:** Summary of clinical studies with metformin, or its combination with EGFR-TKIs or chemotherapeutic agents

Study design/Patient subgroup selection	Diabetic/ Non-diabetic status	Treatment arm(s)	Metformin dosage variation	Targets	PFS and OS	References
Adenocarcinoma patients	Not mentioned	Metformin	Retrospective study, metformin dose not given	CEBPB and PD-L1	Low expression of CEBPB and PD-L1 was associated with better survival probability	[78]
Advanced-stage NSCLC patients with EGFR mutation	Diabetic	Metformin + EGFR-TKI	Retrospective study, metformin dose not given	EGFR mutation	Increase PFS and OS	[74]
Stage IIIB/IV lung adenocarcinoma patients with EGFR mutation	Non-diabetic	Metformin + EGFR-TKI	Started with 500 mg Bid and then reduced to 500 mg daily in patients who experienced drug-related adverse events	EGFR mutation	Longer median PFS and median OS	[98]
Stage IIIB/IV NSCLC with EGFR-ALK wild-type	Both diabetic and non-diabetic	Metformin + Chemotherapy	Started with 500 mg Bid for 1 week, and then increased to 1000 mg Bid if patients tolerated	–	No survival benefit in unselected patients	[96]
Stage IV chemotherapy naïve non-squamous NSCLC patients	Non-diabetic	Metformin + Pemetrexed + Carboplatin	1st cycle started with 500 mg Bid. After a week, increased to 1000 mg first daily dose and 500 mg second dose. From the 2nd cycle, metformin was given 1000 mg Bid	STK11 mutation	No benefit in improving the PFS rate	[99]
Unresected stage IIIA or IIIB NSCLC	Non-diabetic	Metformin + Chemoradiotherapy	Started with 1000 mg daily. In week 2, the metformin dose increased to 1500 mg daily and then escalated to 2000 mg daily in week 3	–	Poor PFS and OS, and higher adverse events	[100]
Unresected stage III NSCLC	Non-diabetic	Metformin + Chemoradiotherapy	Started with 500 mg Bid. In week 2, the metformin dose increased to 500 mg thrice a day	–	No benefit in improving survival	[101]

*NSCLC* non-small cell lung cancer, *EGFR* epidermal growth factor receptor, *Bid* twice a day, *CEBPB* CCAAT-enhancer-binding protein beta, *PD-L1* programmed cell death ligand-1, *STK11* serine/threonine kinase 11, *PFS* progression-free survival, *OS* overall survival, *TKI* tyrosine kinase inhibitor, *ALK* anaplastic lymphoma kinase

### Metformin combination with EGFR-TKIs

Regarding the metformin combination, a retrospective clinical study explored advanced-stage NSCLC patients with T2DM harboring EGFR mutations such as L858R and 19del who received either concurrent metformin or other hypoglycemic drugs with EGFR-TKIs treatment [74]. The data demonstrated that patients who received metformin treatment with EGFR-TKIs exhibited better response, including higher stable disease conditions, progression-free survival (PFS), and overall survival (OS) compared to non-metformin users [74]. Along similar lines, a prospective phase 2 randomized clinical trial was conducted to evaluate and compare the efficacy of a combination of metformin and EGFR-TKIs with EGFR-TKIs alone in stage IIIB-IV lung adenocarcinoma patients harboring an activating EGFR mutation [98]. The EGFR-TKIs included erlotinib hydrochloride, afatinib dimaleate, or gefitinib. The primary outcome was to assess the PFS, and secondary outcomes included objective response rate (ORR), disease control rate, OS, and safety. The data demonstrated that metformin treatment improves the clinical response of first/second-line EGFR-TKIs (i.e., significantly longer median PFS and median OS) compared to the EGFR-TKIs alone group [98]. However, the frequency of grade 3 or 4 adverse events (i.e., diarrhea, rash, nausea, and mucositis) was similar in both treatment groups. The limitations of these findings included the lack of randomization stratification per smoking status, the EGFR mutation profile/EGFR-TKIs, and conducting a non-double-blind study. Overall, these findings indicated that metformin addition could be used as a promising option to overcome EGFR-TKI resistance in NSCLC patients.

### Metformin combination with chemotherapy

Given that metformin in combination with EGFR-TKIs was found to improve the PFS and OS of NSCLC patients, its efficacy was evaluated with chemotherapy regimens. To that end, Lee et al. [96] conducted a randomized phase II clinical trial in chemo-native, EGFR-anaplastic lymphoma kinase (ALK) wild-type, stage IIIB/IV NSCLC patients. The treatment group included metformin combination with chemotherapy (i.e., gemcitabine + carboplatin), and the control group received chemotherapy alone. The patient outcomes included the risk of disease progression and survival. Metformin combination significantly decreased the risk of progression and improved the survival of those squamous cell carcinoma (SqCC) patients who exhibited high fluorodeoxyglucose uptake in SqCC in positron-emission tomography imaging compared to non-SqCC patients [96]. However, no survival benefit was seen in patients whose insulin level was reduced after the metformin combination, or who had hyperinsulinemia. While alterations in *TP53, RAS,* and *PI3K-Akt-mTOR* genes were not predictive of PFS and OS, significantly increased levels of high-density lipoprotein (HDL)-cholesterol in the metformin group were associated with better PFS and OS [96]. Overall, these findings suggested that while metformin combination exerted a synergistic antitumor effect in selected SqCC patients, whose tumors are highly dependent on glucose metabolism, it did not show any survival benefits in unselected non-SqCC NSCLC patients.

Along similar lines, Verma et al. [99] conducted a single-arm phase 2 clinical trial to determine the efficacy of metformin with pemetrexed and carboplatin in treatment-naïve advanced-stage non-squamous NSCL patients. The primary outcome is 6 month PFS, and secondary outcomes are the safety, OS, ORR, proportion, and effect of STK11 mutation (a subgroup who are inherently resistant to immune checkpoint inhibitors) on the rate of 6 month PFS [99]. Out of 26 patients, 25 patients had a median PFS of 4.5 months, and the 6 month PFS rate was 28%. Forty percent of patients experienced partial response, 32% had stable disease, and the ORR was 72%. The mutation analysis was done in only 9 patients, and 2 patients with STK11 mutation had relatively low PFS (< 12 weeks), indicating limitations to precisely draw any conclusions on the efficacy of combination therapy in STK11-mutated patients. Overall, these findings indicated that while the combination therapy was found to be safe and tolerable with common gastrointestinal toxicities such as nausea, vomiting, and diarrhea, no improvement in the 6 month PFS rate was documented [99].

### Metformin combination with chemoradiotherapy

Given that locally-advanced NSCLC (LA-NSCLC) patients experience poor OS with concurrent chemoradiotherapy, Tsakiridis et al. [100] determined the efficacy of metformin in the same context in a multicenter phase 2 randomized clinical trial, termed The Ontario Clinical Oncology Advanced lung Cancer Treatment with Metformin and Chemoradiotherapy (OCOG-ALMERA) study. The metformin was given concurrently with chemotherapy regimens: cisplatin + etoposide, cisplatin + vinorelbine, carboplatin + etoposide, or paclitaxel combined with radiotherapy. The assessment of the proportion of patients who experienced a failure event was the primary outcome, which included disease progression, distant metastases, death, and not following treatment or evaluations within 12 months. [100]. The studies demonstrated treatment failure in 18 out of 26 patients (69.2%) in the metformin arm compared to 12 out of 28 (42.9%) in the control (without metformin) arm. Notably, in the metformin arm, the 1 year PFS and OS rates were 34.8% and 47.4% compared to 63.0% and 85.2% in the control arm. In addition, the adverse events were higher in the metformin (53.8%) compared to the control (25%) arm [100]. The limitations included a lack of double-blinding, placebo control, and limited accrual due to the exclusion of patients with diabetes. Overall, these findings indicated that metformin combination with chemoradiotherapy was associated with poor treatment efficacy and adverse effects. While the underlying mechanisms of treatment failure and associated side effects with metformin are not adequately explored in the manuscript, these findings indicate that metformin should not be used with chemoradiotherapy in LA-NSCLC patients.

Likewise, Skinner et al. [101] conducted an open-label phase 2, NRG-LU001 randomized clinical trial to examine the effects of metformin combination with concurrent chemoradiation and chemoradiation alone in non-diabetic unresected stage III NSCLC patients. The primary outcome was 1-year PFS, and the secondary outcomes included OS, time to local–regional recurrence and metastasis, and adverse events. The data demonstrated no significant differences in the recurrence, metastasis, and adverse events; however, the 1 year PFS and 1 year OS rates in the metformin combination were 51.3% and 80.8% compared to 60.4% and 80.2% in the control arm [101]. The limitations of these findings included: 1) while 63.2% of patients received metformin, only 39% of patients were able to maintain it at the given dose without modifications; and 2) the absence of a placebo group. Overall, these findings suggest that while metformin in combination with concurrent chemoradiation was well tolerated, it did not improve the PFS and OS in NSCLC patients.

The possible reasons/underlying mechanisms of metformin failure in improving chemoradiation efficacy in OCOG-ALMERA and NRG-LU001 clinical trials may be as follows. (1) These trials did not include patients with preexisting diabetes, compared to other trials where improved EGFR-TKIs responses were noted with metformin in diabetic NSCLC patients [74, 98]. (2) Lack of evaluation of genetic and serum biomarker testing in these trials, as NSCLC patients harboring EGFR mutations and increased levels of serum HDL-cholesterol were associated with better PFS and OS in the metformin combination group [74, 96]. While it is questionable whether metformin should further be explored with chemoradiotherapy, emerging evidence from preclinical studies and phase 1b trial indicates that, in addition to EGFR-TKIs, metformin may augment immune-based therapy such as anti-PD1 and anti-PD-L1 immune checkpoint inhibitors [102–105].

Notably, the common inconsistencies between preclinical and clinical studies that affect the overall responses of metformin combination therapy are summarized in Table 3.

**Table 3:** Disparities between preclinical studies and clinical trials with metformin combination regimens

Parameters	Preclinical studies	Clinical trials	Translational inconsistencies
Metformin dose variation	Often 1 – 10 mmol/L in vitro and high doses in animal models	500 – 2000 mg/d	Efficacy may not be achievable in human tumors
Treatment schedule	Started before, soon after tumor implantation, or during early tumor growth	Started in the advanced stage or metastatic disease	Early intervention in preclinical studies may enhance treatment response
Schedule for drug combination	Precisely timed at controlled intervals	Timing is based on patient tolerance and clinical logistics	Synergy is impacted if timing varies
Tumor model	Homogeneous cell lines, xenografts, or genetically-engineered mouse models	Heterogeneous tumors with several driver mutations	Higher genetic variability in human responses
Tumor microenvironment (TME) and immunity	Often lacks immune and stromal components	Consists of immune and stromal components	Human TME can hinder drug efficacy
State of health	Healthy, young, or non-diabetic	Often older with comorbid conditions (e.g., diabetes)	Comorbid conditions can affect drug tolerability
Study control	Tightly controlled environment and diet	Variable environment, diet, and medications	Variability in patients adds confounding factors
Common endpoints/ outcome measures	Suppression of tumor volume/biomarker changes in a short period of time	PFS and OS over months to years	Patient outcomes are often challenging to improve significantly

*PFS* progression-free survival, *OS* overall survival

## Conclusion, limitations, and future perspectives

Several in vitro and in vivo studies have confirmed the potential anti-cancer activity of metformin against NSCLC. Metformin targets various activities of tumor cells, including proliferation, migration, invasion, and angiogenesis, to induce cell cytotoxicity, cell cycle arrest, and apoptosis, resulting in decreased tumor growth and metastasis as documented in in vitro, in vivo, and ex vivo studies. The ability of metformin to target multiple cell signaling pathways provides a rationale for its exploration with other therapeutic agents for the treatment of NSCLC. Several classes of anti-cancer agents have been evaluated with metformin and found to exert synergistic antitumor effects. Notably, metformin has also been used to overcome drug resistance mechanisms. Importantly, metformin in combination with EGFR-TKIs has been shown to improve the clinical outcomes in NSCLC patients. Moreover, metformin can be used as a promising combination approach to target other potential signaling cascades involved in tumor development and/or impede the efficacy of therapeutic agents [106–108].

While metformin combination with chemotherapeutic agents resulted in synergistic antitumor effects in experimental in vitro and in vivo models, it did not show preferable outcomes in terms of PFS and OS in clinical settings, which remain the ongoing challenges and limitations to be explored in future research. Moreover, metformin’s side effects, such as gastrointestinal issues (e.g., nausea and diarrhea), B12 deficiency, and lactic acidosis, which are found to be associated with higher dosing regimens and longer treatment duration, also affect its clinical translation [109–111]. Notably, these deficiencies can cause anemia and peripheral neuropathy, which have significant overlap with the side effects of chemotherapy or radiotherapy that lung cancer patients experience. Furthermore, compared to other repurposed drugs such as statins and aspirin, which are associated with reduced lung cancer incidence/mortality, only a weak association was noticed between metformin and overall survival of lung cancer patients [100, 101, 112–116]. These studies suggest that greater attention should be given to dosing optimization and patient stratification (e.g., with characteristics similar to those of lung cancer patients who benefited from statin or aspirin) to explore metformin’s effect in combination with other therapeutic agents.

Along similar lines, biomarkers guide lung cancer treatment by predicting therapy outcomes or emerging resistance [117]. For example, EGFR mutations (exon 19 deletion or L858R) predict responses to osimertinib (i.e., 46% disease-free survival in stage IIA-IIIA NSCLC patients) [118]. Thus, the identification of potential biomarkers and their implications in selecting a subgroup of NSCLC patients who could benefit from metformin treatment is important to determine the best combination approaches, including strategies to avoid adverse events.

In addition, the emerging trends indicate that metformin analogs such as phenformin show promising and stronger anti-cancer effects than metformin, or exert synergistic efficacy with other therapeutic agents in experimental NSCLC models [119, 120]. Moreover, nanotechnology-based delivery of metformin via hyaluronan-coated mesoporous silica nanoparticles or PEGylated niosomal nanoparticles has not only shown an enhanced cellular uptake and cytotoxicity compared to free metformin, but also increased synergy with other drugs [121, 122]. Thus, these new strategies could also be explored in enhancing metformin-based combination approaches for NSCLC treatment. Taken together, metformin should be evaluated as a novel therapeutic strategy with careful consideration of other anti-cancer agents in clinical trials for the treatment of NSCLC.

## Data Availability

Not applicable.

## Abbreviations

2-dDG: 2-deoxy D-glucose
Akt: Protein kinase B
AMP: Adenosine monophosphate
AMPK: AMP activated protein kinase
ATP: Adenosine triphosphate
ASS: Acetyl-coA carboxylase
CDKN1B: Cyclin-dependent kinase inhibitor 1B
CEBPB: CCAAT-enhancer-binding protein beta
Deptor: DEP domain-containing mTOR-interacting protein
4E-BP1: Eukaryotic translation initiation factor 4E binding protein 1
EGFR: Epidermal growth factor receptor
ERK: Extracellular signal-regulated kinase
FADD: Fas-associated death domain
GLP-1: Glucagon-like peptide-1
HER2: Human epidermal growth factor receptor 2
HIF-1α: Hypoxia-inducible factor-1α
HO-1: Heme oxygenase-1
IGF: Insulin growth factor
IGF-1R: IGF-1 receptor
KRAS: Kirsten rat sarcoma viral oncogene homolog
LAP: Liver-enriched transcriptional activator protein
LDH: Lactose dehydrogenase
LKB1: Liver kinase B1
LIP: Liver-enriched inhibitory protein
MAPK: Mitogen-activated protein kinase
MEK: Mitogen-activated protein kinase kinase
mTORC1: Mammalian target of rapamycin complex 1
NF-κB: Nuclear factor-kappa B
Nrf2: Nuclear factor erythroid-2 related factor 2
NSCLC: Non-small cell lung cancer
OCT1: Organic cation transporter 1
OS: Overall survival
PARP: Poly(ADP-ribose) polymerase
PD-1: Programmed cell death protein-1
PD-L1: Programmed cell death-ligand 1
PDX: Patient-derived xenograft
PFS: Progression-free survival
PI3K: Phosphatidylinositol 3-kinase
PRAS40: Proline-rich protein kinase B substrate of 40 kD
RAF: Rapidly accelerated fibrosarcoma
RHEB: Ras homolog enriched in brain
RAS: Rat sarcoma viral oncogene homolog
ROS: Reactive oxygen species
RTKs: Receptor tyrosine kinases
SCLC: Small cell lung cancer
S6K1: Ribosomal protein S6 kinase B1
SqCC: Squamous cell carcinoma
SOS: Son of sevenless
STK11: Serine/threonine kinase 11
STING: Stimulator of interferon genes
TBK1: TANK-binding kinase 1
TKIs: Tyrosine kinase inhibitors
TRAIL: Tumor necrosis factor-related apoptosis-inducing ligand
TRF3: TATA-box-binding protein-related factor 3
TSC: Tuberous sclerosis protein
